# Comparison of gene expression profiles and related pathways in chronic thromboembolic pulmonary hypertension

**DOI:** 10.3892/ijmm.2013.1582

**Published:** 2013-12-10

**Authors:** SONG GU, PIXIONG SU, JUN YAN, XITAO ZHANG, XIANGGUANG AN, JIE GAO, RUI XIN, YAN LIU

**Affiliations:** Department of Cardiac Surgery, Beijing Chao-Yang Hospital, Capital Medical University, Beijing 100020, P.R. China

**Keywords:** chronic thromboembolic pulmonary hypertension, gene, microarray, Gene Ontology, signaling pathway

## Abstract

Chronic thromboembolic pulmonary hypertension (CTEPH) is one of the main causes of severe pulmonary hypertension. However, despite treatment (pulmonary endarterectomy), in approximately 15–20% of patients, pulmonary vascular resistance and pulmonary arterial pressure continue to increase. To date, little is known about the changes that occur in gene expression in CTEPH. The identification of genes associated with CTEPH may provide insight into the pathogenesis of CTEPH and may aid in diagnosis and treatment. In this study, we analyzed the gene expresion profiles of pulmonary artery endothelial cells from 5 patients with CTEPH and 5 healthy controls using oligonucleotide microarrays. Bioinformatics analyses using the Gene Ontology (GO) and KEGG databases were carried out to identify the genes and pathways specifically associated with CTEPH. Signal transduction networks were established to identify the core genes regulating the progression of CTEPH. A number of genes were found to be differentially expressed in the pulmonary artery endothelial cells from patients with CTEPH. In total, 412 GO terms and 113 pathways were found to be associated with our list of genes. All differential gene interactions in the Signal-Net network were analyzed. JAK3, GNA15, MAPK13, ARRB2 and F2R were the most significantly altered. Bioinformatics analysis may help gather and analyze large amounts of data in microarrays by means of rigorous experimental planning, scientific statistical analysis and the collection of complete data. In this study, a novel differential gene expression pattern was constructed. However, further studies are required to identify novel targets for the diagnosis and treatment of CTEPH.

## Introduction

Chronic thromboembolic pulmonary hypertension (CTEPH) is one of the main causes of severe pulmonary hypertension. CTEPH is characterized by the presence of unresolved thromboemboli associated with fibrous stenosis in the proximal pulmonary arteries. Diagnosis is usually made in the advanced stages of the disease when pulmonary vascular resistance (PVR) has increased by 5- to 10-fold. This increase in PVR resistance results in pulmonary hypertension and progressive right heart failure. It may be caused by a single or recurrent pulmonary embolism and/or the local formation of thrombi. The proximal location of pulmonary artery obliteration is the main feature observed in patients with CTEPH that differs from pulmonary arterial hypertension (PAH) (1).

Depending on the localization and extent of proximal thrombotic material, a pulmonary endarterectomy (PEA) may be necessary (2). Approximately half of the pulmonary blood flow dynamics can return to normal levels following PEA. However, in approximately 15–20% of patients, PVR and pulmonary arterial pressure (PAP) continue to rise, thus increasing the mortality rate by up to 4–5% (3). It has been suggested that the reason for the development of the persistent occlusion of the pulmonary artery is a misguided thrombus resolution triggered by infection (4), inflammation (5), autoimmunity, malignancy (6) and/or endothelial dysfunction due to a high presence of phospholipid antibodies and lupus anticoagulants (7,8) rather than prothrombotic factors.

A number of factors, such as C-reactive protein (CRP) (9), endothelin-1 and von Willebrand factor (10) may participate in the pathophysiology of pulmonary hypertension. However, there are hundreds of implicated genomic loci with heterogeneous functions. As a result, there is difficulty in understanding the mechanisms by which this diverse genetic susceptibility translates to a common clinical phenotype. The advent of genome-wide technologies, such as gene expression microarray, has made it possible to achieve a comprehensive view of the alterations in gene expression occurring in CTEPH. In the present study, we used bioinformatics to analyze the differences in gene expression between CTEPH and normal tissue. The different Gene Ontology (GO) terms and pathways revealed the most important mechanisms and candidate genes involved in the development of CTEPH; our data may aid in the development of more individualized treatment regimens according to the genetic characteristics of individual patients.

## Patients and methods

### Patients

Five consecutive patients with CTEPH were enrolled in this study from the Beijing Chao-Yang Hospital, Capital Medical University, Beijing, China between June 2012 and February 2013. The study was approved by the relevant ethics commitee and all patients provided written consent to participate in this study. All patients were examined using lung ventilation and perfusion scans, right-heart catheterization and pulmonary angiography to confirm the diagnosis. Patients with CTEPH were defined as those having a mean pulmonary arterial pressure (mPAP) of ≥25 mmHg with normal wedge pressure (≤12 mmHg) who had dyspnea on exertion during a period of >6 months on effective anticoagulation. In addition, lung perfusion scans were performed to demonstrate a segmental or larger defect concomitant with a normal ventilation scan (11,12). Finally, chronic thromboembolic findings were confirmed on pulmonary angiography (13). Five healthy controls (donors for lung transplants) were also included.

### Microarray analysis

For Affymetrix microarray profiling, total RNA of CTEPH and normal tissues was isolated using TRIzol reagent (Invitrogen, Burlington, ON, Canada) and purified using the RNeasy Mini kit (Qiagen, Hilden, Germany), including a DNase digestion treatment. RNA concentrations were determined by absorbance (A) at 260 nm and quality control standards were A260/A280 = 1.8–2.1, using a NanoDrop 2000 spectrophotometer (Thermo Scientific, Wilmington, DE, USA).

cDNA of pulmonary artery endothelial cells from patients with CTEPH or the normal controls was hybridized to Human Gene 2.0 ST GeneChip^®^ arrays (Affymetrix, Inc., Santa Clara, CA, USA) according to the manufacturer’s instructions. Affymetrix^®^ Expression Console Software (version 1.2.1) was used for microarray analysis. Raw data (CEL files) were normalized at the transcript level using the robust multi-average method (RMA workflow). The median summarization of transcript expressions was calculated. Gene-level data was then filtered to include only those probe sets that are in the ‘core’ metaprobe list, which represent RefSeq genes.

### Analysis of differential gene expression

The RVM t-test was applied to filter the differentially expressed genes for the control and experimental group, as the RVM t-test can raise degrees of freedom effectively in the cases of small samples. After the analysis of differentially expressed genes and false discovery rate (FDR) analysis, we selected the differentially expressed genes according to the P-value threshold. A value of P<0.05 was considered to indicate a statistically significant difference, as previously described (14–16). The data of differentially expressed genes were subjected to unsupervised hierarchical clustering (Cluster 3.0) and TreeView analysis (Stanford University, Stanford, CA, USA).

### GO analysis

GO analysis was applied to determine the main functions of the differential expression genes according to the GO database, which is the key functional classification of NCBI, which organizes genes into hierarchical categories and identifies the gene regulatory network on the basis of biological process and molecular function (17,18).

Specifically, a two-sided Fisher’s exact test and χ^2^ test were used to classify the GO categories, and the FDR (19) was calculated to correct the P-value; the smaller the FDR, the smaller the error in judging the P-value. The FDR was defined as:

$$FDR=1-\frac{N_{k}}{T}$$

where *N_k_* refers to the number of Fisher’s test P-values less than χ^2^ test P-values. We computed P-values for the GO terms of all the differentially expressed genes. Enrichment analysis provides a measure of the significance of the function: as the enrichment increases, the corresponding function is more specific, which helps us to find those GO terms with a more concrete functional description in the experiment. Within the significance category, the enrichment Re was given by Re = (*n_f_*/*n*)/(*N_f_*/*N*), where *n_f_* is the number of flagged genes within the particular category, *n* is the total number of genes within the same category, *N_f_* is the number of flagged genes in the entire microarray, and *N* is the total number of genes in the microarray, as previously described (20).

### GO-map

GO-map analysis is the interaction network of the significant GO terms of the differentially expressed genes, and was carried out to integrate the associations between these GO terms by outlining the interactions of related GO terms and summarizing the functional interactions of the differentially expressed genes in diseases (18,20).

### Pathway analysis

Pathway analysis was used to identify the common pathways associated with the differentially expressed genes according to the KEGG, BioCarta and Reatome databases. We used the Fisher’s exact test and χ^2^ test to identify the significant pathways, and the threshold of significance was defined by a P-value and FDR. The enrichment Re was calculated with the equation described above, as previously described (21–23).

### Path-Net

Path-Net is the interaction network of the most common pathways associated with the differentially expressed genes, and was built according to the interaction among pathways of the KEGG database to determine the interactions among the significant pathways directly and systemically. It identified the common pathways associated with the differentially expressed genes, as well as the mechanisms behind the activation of a certain pathway (22).

### Signal-Net analysis

Using java that allows users to build and analyze molecular networks, network maps were constructed. For instance, if there is confirmative evidence that two genes interact with each other, an interaction edge is assigned between the two genes. The considered evidence is the source of the interaction database from KEGG. Networks are stored and presented as graphs, where nodes are mainly genes (protein, compound, etc.) and edges represent relation types between the nodes, e.g., activation or phosphorylation. The graph nature of Networks peaked our interest to investigate them with powerful tools implemented in R.

In order to investigate the global network, we computationally identified the most important nodes. To this end, we determined the connectivity (also known as the degree) defined as the sum of connection strengths with the other network genes:

$${\text{K}}_{i}=\sum_{u\neq i}a_{ui}$$

In gene networks, the connectivity measures how well a gene correlates with all other network genes. For a gene in the network, the number of source genes of a gene is called the indegree of the gene and the number of target genes of a gene is its outdegree. The character of genes is described by betweenness centrality measures reflecting the importance of a node in a graph relative to other nodes. For a graph G:(V,E) with n vertices, the relative betweenness centrality *C’_B_(v)* is defined by:

$$C_{B}^{\prime }(v)=\frac{2}{n^{2}-3n+2}\sum_{\begin{array}{l} s\neq v\neq t\in v\\ s\neq t \end{array}}\frac{\sigma_{st}(v)}{\sigma_{st}}$$

where *σ_st_* is the number of shortest paths from s to t, and *σ_st_(v)* is the number of shortest paths from s to t that pass through a vertex v (24–28).

### Data analysis

Numerical data are presented as the means ± standard deviation (SD). Differences between means were analyzed using the Student’s t-test. All statistical analyses were performed using SPSS 13.0 software (SPSS, Inc., Chicago, IL, USA).

## Results

### Clinical characteristics of the two sample groups

The characteristics of the 5 consecutive patients with CTEPH (3 male, 2 female) enrolled in the study are presented in Table I. In addition, tissue from healthy volunteers was obtained from donors of lung transplants and matched to the patients with CTEPH. All patients with CTEPH did not have lung cancer and underwent anti-vitamin K treatment using warfarin. The median D-dimer level of the patients with CTEPH was 0.499 μg/ml (range, 0–1.32 μg/ml).

### CTEPH-related differential gene expression profiles

**Figure 1:**
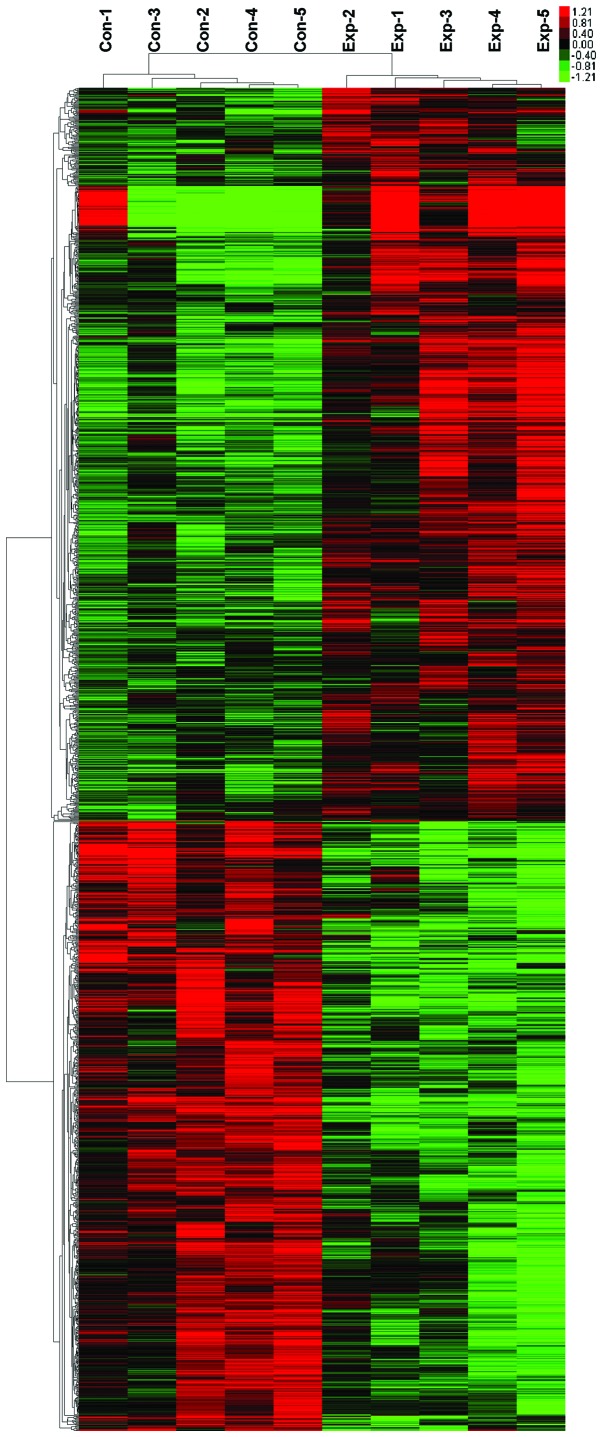
Unsupervised classification of chronic thromboembolic pulmonary hypertension (CTEPH) and healthy control samples based on gene expression profiling. Classification of 10 pulmonary artery endothelial cell samples using the differentially expressed 2098-probe sets. Expression data are depicted as a data matrix where each row represents a gene and each column represents a sample. Expression levels are depicted according to the color scale shown at the top. Red and green indicate expression levels above and below the median, respectively. The magnitude of deviation from the median is represented by the color saturation.

Genome-wide transcriptional profiling of tumors has demonstrated that extensive gene expression occurs after the formation of CTEPH. To investigate the possible changes in gene expression, microarray analysis was used to analyze the gene expression profiles in the patients with CTEPH and normal tissue groups and 1,614 genes with statistically significant changes in expression were identified. Of these, 880 genes were upregulated in the CTEPH samples and 734 were downregulated. Ten genes that were the most significantly upregulated or downregulated according to their P-values are listed (Tables II and III). Hierarchical clustering revealed systematic variations in the expression of genes between the two groups (Fig. 1). The results demonstrated that these differential probes could clearly separate the two groups from the whole samples.

### GO analysis of differentially expressed genes in CTEPH and GO map

**Figure 2:**
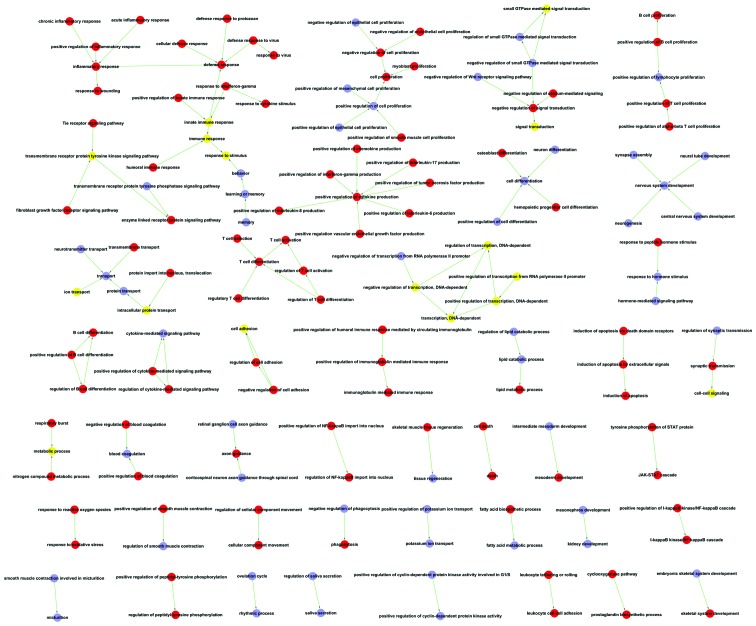
GO map of significant differentially expressed genes: red circles represent the GO categories of upregulated genes, lavender circles represent the GO categories of downregulated genes and yellow circles represent the GO categories associated with both upregulated and downregulated genes.

Significant progress in data mining has provided a wide range of bioinformatics analysis options. For example, GO, which has been proven to be extremely useful for the mining of functional and biological significance from very large datasets (17,18), can produce a controlled vocabulary used for dynamic maintenance and interoperability between genome databases. GO analysis of the differentially expressed genes in the two groups was performed. A total of 235 GO items associated with upregulated genes and 177 GO items associated with downregulated genes in the two groups were obtained (Table IV). For the GO items listed in the table, a GO map was constructed to further define the results of GO analysis (Fig. 2). In the GO map, items regarding defense response were the most common between the two groups, suggesting that the formation of thrombi was mainly caused by tissue response to various stimuli, such as inflammation and immune response. Furthermore, items regarding cell proliferation, signal transduction and cytokine production were also very common (high enrichment score). All these items indicated that apart from the traditional knowledge of the role of thrombosis in the development of CTEPH, other mechanisms, such as signaling pathway and cytokines may also play an important role in its pathogenesis.

### Pathway analysis of differentially expressed genes in CTEPH and Path-Net

**Figure 3:**
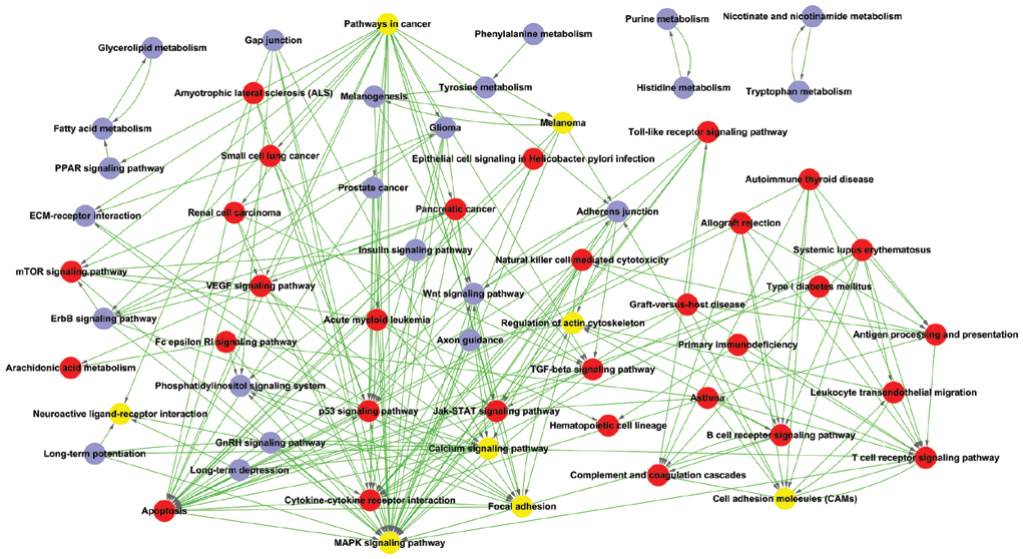
Path-Net network of significantly differentially expressed genes: red circles represent the pathways associated with upregulated genes, lavender circles represent the pathways associated with downregulated genes and yellow circles represent the pathways associated with both up- and downregulated genes.

To determine the involvment of signal transduction pathways in CTEPH, related pathways were analyzed according to the functions and interactions of the differentially expressed genes. By using pathway analysis with the threshold of significance defined on the basis of P<0.05, a great number of significant pathways was found (Tables V and VI). The high enriched pathways targeted by overexpressed genes were involved in cytokine-cytokine receptor interaction, leishmaniasis and cell adhesion molecules (CAMs). By contrast, significant pathways corresponding to underexpressed genes appeared to be responsible for focal adhesion, neuroactive ligand-receptor interaction and arrhythmogenic right ventricular cardiomyopathy (ARVC). However, for the pathways listed which seemed to not be relevant to CTEPH, Path-Net was used to analyze the different pathways to identify the most important ones (Fig. 3). In Path-Net, the MAPK signaling pathway and apoptosis had the largest enrichment degree, which suggests that they are not the most significant variation pathways but participate in pathway regulation. Identifying these pathways may help us regulate the related pathways and control the development of CTEPH.

### Signal transduction networks in CTEPH

**Figure 4:**
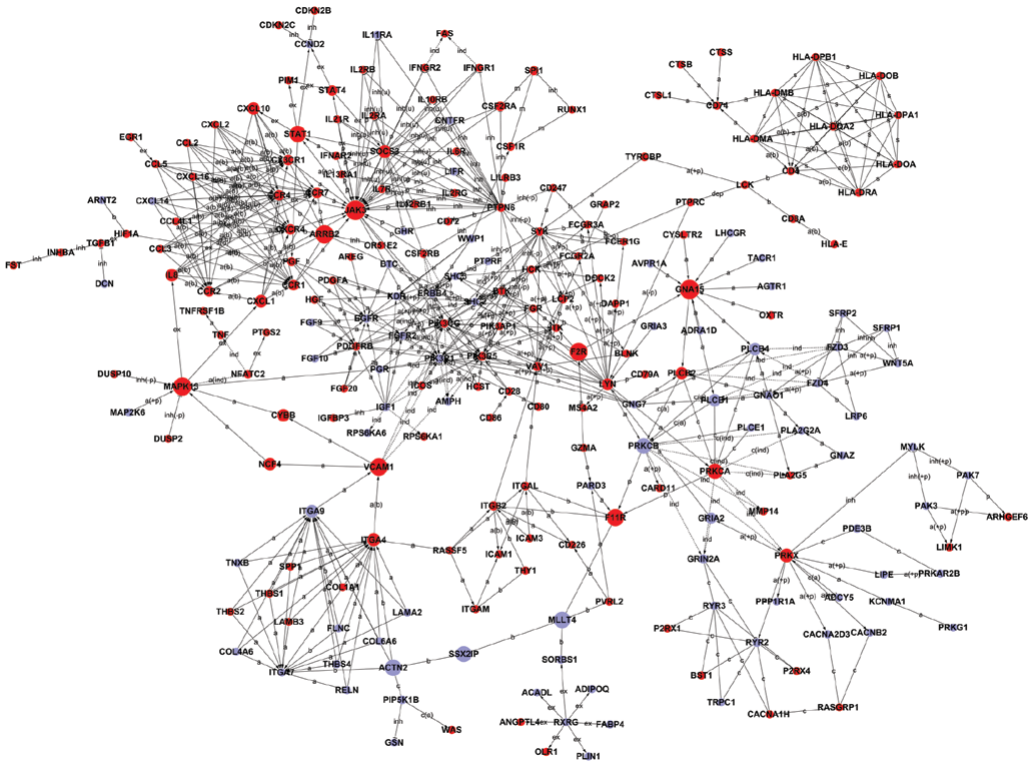
Signal transduction networks of CTEPH-related genes. Circles represent genes, red circles represent upregulated genes, and blue circles represent downregulated genes. Arrows represent the activation of (a); straight line represents combinations; dotted line represents indirect effects; a, represents activation; ex, represents gene expression; b, represents binding; c, represents compound; ind, represents indirect effects; inh, represents inhibition; u, represents ubiquination, s, represents state change; detailed annotation listed in Table VII.

According to the literature and experimental records in the databases, 440 (276+164) genes appearing in previous 113 (62+51) pathways were collected and a diagram of the gene interaction network was drawn up based on these genes (Fig. 4). The total number of genes in the network was 232, and the particular associations between them are listed in Table VII. In the network, cycle nodes represent genes, and the edges between two nodes represent the interactions between genes, which were quantified by betweenness centrality. Betweenness centrality within the network which contains both the direct regulation by degree and the signal transmitting between the genes represents the size of the cycle node. The higher the betweenness centrality, the more common the gene within the network. The clustering coefficient can be used to estimate the complexity of interactions among genes that neighbor the core gene with the exception of core gene participation. The lower the clustering coefficient, the more independent of the core gene is the interaction among genes in the neighborhood of the core gene. Janus kinase 3 (JAK3), guanine nucleotide binding protein (G protein), alpha 15 (Gq class) (GNA15), mitogen-activated protein kinase 13 (MAPK13), arrestin, beta 2 (ARRB2) and coagulation factor II (thrombin) receptor (F2R) were the five main central genes identified by betweenness centrality.

## Discussion

Several clinical and therapeutic factors have been reported as significant to the occurrence of CTEPH. The pathophysiology of CTEPH remains incompletely understood. In most cases it is associated with a history of acute venous thromboembolism (29); however, in a small percentage of patients, thrombi do not resolve after an acute event and the reasons for this are unclear. The current knowledge is based on a triad of enhanced thrombosis (7,30), disturbed thrombolysis (31–33) and inflammation (34). A number of novel prognostic factors, such as cytological features, standard karyotyping, fluorescence *in situ* hybridization, centromeric probes, single nucleotide polymorphism and gene expression profiling have been investigated. Using the advanced and inexpensive technique of microarray, new genes which may affect the development of CTEPH can be identified.

In this study, to investigate variations in gene expression profiles and signaling pathways in CTEPH, 10 samples were divided into a normal (control) and CTEPH group to identify CTEPH-related differentially expressed genes. The expression of the upregulated genes was higher in the CTEPH group compared with the control group. Gene chips have become a useful tool for studying the development and progression of tumors owing to the high-throughout, but it remains difficult to predict patients with CTEPH, mainly due to the high number of variations in CTEPH and the great challenge in interpreting numerous complex data produced by the microarray (35) and determining the main responsible genes. The present study made use of bioinformatics the method to analyze functions and pathways of the differentially expressed genes, and further clarified their biological significance, and defined the key genes that affect the development of CTEPH.

More than 1,600 genes were differentially upregulated or downregulated in CTEPH in this study. The first upregulated differentially expressed gene in CTEPH, oxidized low density lipoprotein (lectin-like) receptor 1 (OLR1), has been studied in several cardiovascular diseases, such as atherosclerosis for its polymorphisms (36), and recently Wynants *et al* found that it is highly expressed CTEPH (5). The second most significantly upregulated gene, intereukin (IL)8, has been found to be associated with hemodynamic instability following PEA in patients with CTEPH (37). The role of the third most upregulated gene, secreted phosphoprotein 1 (SPP1), which encodes osteopontin, in CTEPH is poorly understood. Most of the genes that were strongly downregulated showed close associations with tumors, chemokines and lipids, including the gene for CXCL14, which is a type of chemokine ligand involved in the regulation of tumors (38,39). Heparanase 2 (HPSE2) encodes a specific enzyme and is associated with urofacial syndrome (40). None of these genes were found to be associated with CTEPH. As the sample number we used in this study was limited, we tried to investigate the microarray results using another method.

GO is widely recognized as the premier tool for the organization and functional annotation of molecular aspects (41). GO analysis was used to interpret each GO of the differentially expressed genes and analyze it statistically. By using the criteria of P<0.05, significant GO items and genes involved were identified. Guo *et al* used GO analysis to analyze miRNA microarrays and found that miR-15b and miR-16 may be indispensable for apoptosis by targeting Bcl-2 (42). GO terms regarding inflammatory response play an important role in CTEPH; a number of studies have reported CRP as a predictor of adverse outcome in pulmonary arterial hypertension (43), and IL-6-mediated systemic inflammatory cascades may also be involved in the regulation of peripheral vascular tone following pulmonary thromboendarterectomy (PTE) (44). Quarck *et al* reported that a proliferative phenotype of pulmonary arterial smooth muscle cells and endothelial cells contributed to proximal vascular remodeling in CTEPH (45). A number of studies have proven that patients with CTEPH can generate a pronounced inflammatory response with the release of pro-inflammatory and anti-inflammatory cytokines (44,46). Therefore, we hypothesized that the functions of other items listed may play a role in CTEPH, which has not been elucidated yet.

GO analysis is a classical method used to annotate gene function but is still inexact in some fields. Pathway analysis can reveal the distinct biological process and identify significant pathways that differentially expressed genes participate in; based on this, we can have a comprehensive understanding as to the interactions of genes, functions that they participate in and associations between up- and down-stream pathways, and identify genes involved in these significant pathways. The concordance of the MAPK signaling pathway, cytokine-cytokine receptor interaction and apoptosis with the GO terms confirmed their critical role in CTEPH. Wei *et al* demonstrated that JNK is a critical molecule in 5-HT-induced pulmonary artery smooth muscle cell (PASMC) proliferation and migration and may act at an important point for crosstalk of the MAPK and phosphoinositide 3-kinase (PI3K) pathways (47); however, to our knowledge, there is no study available on the role of the MAPK signaling pathway in CTEPH. Numerous studies have proven that focal adhesion and cytokine participate in the process of vascular remodeling, which is an important characteristic of CTEPH (48,49). A previous study on the role of CRP in proximal pulmonary endothelial cells and smooth muscle cells also demonstrated the effects of cell adhesion molecules on CTEPH (5). Since CTEPH is still a rare disease worldwide, information on the signaling pathways associated with its development is limited. We hypothesized that the other seemingly irrelevant pathways may play a role in CTEPH. However, this requires further investigation. Pathway analysis revealed equally important roles and functions as GO analysis.

In the investigation of genes involved in significant GO terms and pathways, 232 genes in common were found that may affect the development of CTEPH. JAK3 is an enzyme in the janus kinase family and has been implicated in cell signaling processes important in cancer and immune-inflammatory diseases (50). Recently, it was found to improve myocardial vascular permeability (51); however, its role in CTEPH remains unelucidated. The functions of GNA15 and ARRB2 have been less frequently reported. MAPK13 mainly participates in cholangiocarcinoma and increases cell migration; it may play a similar role in CTEPH (52). F2R, also known as PAR-1, affects vascular remodeling in the small intestine (53). Vascular cell adhesion molecule 1 (VCAM1) has been reported to modulate blood vessel endothelial cell-leukocyte interactions and increase the strength of cell adhesion (54). Although their functions have not been fully investigated, a number of genes may play a role in the development of CTEPH. Based on these data, further studies on the expression of these genes and protein functions are required using a greater sample number and using methods, such as reverse transcriptase-polymerase chain reaction and western blot analysis; moreover, the regulatory functions of the identified genes and proteins also requires investigation. Further studies may help to improve the clinical diagnosis and treatment of patients with CTEPH.

In conlcusion, the results presented in our study suggest that differences in gene expression exist between CTEPH and normal samples. These genes encode proteins involved in different GO items and signaling pathways, the disruption of which can affect the development of CTEPH. Several genes, such as JAK3, GNA15, MAPK13, F2R and VCAM1 provide potential candidates for distinguishing between CTEPH from healthy individuals in the future. This distinction may aid in the diagnosis and prevention of CTEPH, based on the different features.

## Figures and Tables

**Table I:** Clinical characteristics of the patients in this study.

Sample	No.	Age (years) mean ± SD	Gender (M/F)	mPAP (mmHg) median (range)	PVR (dyne·s·cm^−5^)	6 WMT
Healthy controls	5	35.3±10.6	2/3	-	-	-
CTEPH patients	5	38.2±14.7	2/3	55 (33–78)	1075.4	454.7

mPAP, mean pulmonary arterial pressure; PVR, pulmonary vascular resistance; CTEPH, chronic thromboembolic pulmonary hypertension; 6 WMT, 6-min walk test.

**Table II:** Most significantly upregulated genes.

Gene symbol	P-value	Geom mean of intensities in CTEPH group	Geom mean of intensities in healthy controls	Fold-change
PTGS2	1.25E-05	155.56	38.18	4.07
TBX15	2.35E-05	95.07	23.66	4.02
FMO3	3.00E-05	120.33	16.06	7.49
LRRC32	3.03E-05	445.65	151.99	2.93
FAM100B	3.87E-05	181.47	92.94	1.95
NEIL3	4.62E-05	16.92	10	1.69
LOC100507286	8.50E-05	33.52	10.1	3.32
LY75	9.46E-05	166.81	70.72	2.36
ITIH3	1.27E-04	79.24	15.24	5.2
TRPV2	1.29E-04	45.02	24.8	1.82

**Table III:** Most significantly downregulated genes.

Gene symbol	P-value	Geom mean of intensities in CTEPH group	Geom mean of intensities in healthy controls	Fold-change
CHRDL1	5.00E-07	11.14	344.38	0.032
FREM1	1.00E-06	13.58	69.85	0.19
BNC2	1.20E-06	33.6	92.91	0.36
ACADL	1.30E-06	13.98	72.87	0.19
UNC13C	3.10E-06	10	27.03	0.37
NCAM1	3.20E-06	17.13	75.75	0.23
PRUNE2	3.30E-06	221.46	706.72	0.31
KLHDC5	4.50E-06	25.88	57.87	0.45
FERMT2	7.40E-06	222.13	409.8	0.54
FAM198A	7.60E-06	24.03	48.54	0.5

**Table IV:** GO items and enrichment scores.

GO name	Enrichment score
T cell selection	34.56136364
T cell activation via T cell receptor contact with antigen bound to MHC molecule on antigen presenting cell	34.56136364
Cell surface pattern recognition receptor signaling pathway	34.56136364
Dicarboxylic acid transport	34.56136364
Cyclooxygenase pathway	34.56136364
Positive regulation of mast cell activation	34.56136364
Regulatory T cell differentiation	34.56136364
Positive regulation of interleukin-10 biosynthetic process	34.56136364
Positive regulation of interleukin-4 biosynthetic process	34.56136364
RNA destabilization	34.56136364
Negative regulation of calcium-mediated signaling	34.56136364
Regulation of cytokine-mediated signaling pathway	23.04090909
Positive regulation of immunoglobulin mediated immune response	23.04090909
Protein deglycosylation	23.04090909
Detection of biotic stimulus	23.04090909
Regulation of smooth muscle cell migration	23.04090909
Viral envelope fusion with host membrane	23.04090909
Lipoxygenase pathway	23.04090909
Response to vitamin B3	23.04090909
Regulation of NF-kappaB import into nucleus	23.04090909
Positive regulation of granulocyte macrophage colony-stimulating factor biosynthetic process	23.04090909
Regulation of cellular component movement	23.04090909
Adhesion to symbiont	23.04090909
Nucleotide-binding oligomerization domain containing 1 signaling pathway	23.04090909
Connective tissue replacement involved in inflammatory response wound healing	20.73681818
Response to peptidoglycan	20.73681818
Regulation of B cell differentiation	20.73681818
Regulation of T cell activation	20.73681818
Nucleotide-binding oligomerization domain containing 2 signaling pathway	20.73681818
Positive regulation of alpha-beta T cell proliferation	18.85165289
Chronic inflammatory response	17.28068182
Regulation of T cell differentiation	17.28068182
Platelet activating factor biosynthetic process	17.28068182
Tyrosine phosphorylation of STAT protein	17.28068182
Pyridine nucleotide biosynthetic process	17.28068182
Regulation of cholesterol transport	17.28068182
Negative regulation of collagen biosynthetic process	17.28068182
Very-low-density lipoprotein particle clearance	17.28068182
Positive regulation of interferon-alpha biosynthetic process	17.28068182
Negative regulation of follicle-stimulating hormone secretion	17.28068182
Antigen processing and presentation of exogenous peptide antigen via MHC class II	14.81201299
Positive regulation of interleukin-2 biosynthetic process	14.23114973
Negative regulation of signal transduction	14.13873967
Chronological cell aging	13.82454545
Membrane raft polarization	13.82454545
Response to molecule of fungal origin	13.82454545
Leukotriene production involved in inflammatory response	13.82454545
Macrophage derived foam cell differentiation	13.82454545
Membrane to membrane docking	13.82454545
Negative regulation of granulocyte differentiation	13.82454545
Tie receptor signaling pathway	13.82454545
Positive regulation of hair follicle development	13.82454545
Positive regulation of chemokine production	12.96051136
Positive regulation of macrophage chemotaxis	12.96051136
Lymphocyte chemotaxis	12.96051136
Negative thymic T cell selection	11.52045455
Negative regulation of blood vessel endothelial cell migration	11.52045455
Positive thymic T cell selection	11.52045455
Positive regulation of humoral immune response mediated by circulating immunoglobulin	11.52045455
Positive regulation of cytokine-mediated signaling pathway	11.52045455
Positive regulation of natural killer cell mediated cytotoxicity directed against tumor cell target	11.52045455
Negative regulation of lipid storage	11.52045455
Death	11.52045455
Cellular response to nutrient	11.52045455
Positive regulation of actin filament bundle assembly	11.52045455
Very-low-density lipoprotein particle assembly	11.52045455
Positive regulation of T-helper 1 cell differentiation	11.52045455
Myoblast proliferation	11.52045455
Negative regulation of focal adhesion assembly	11.52045455
T-helper 1 type immune response	10.63426573
Enzyme linked receptor protein signaling pathway	10.36840909
Negative regulation of plasminogen activation	10.36840909
Leukocyte tethering or rolling	10.36840909
Cell recognition	9.874675325
Positive regulation of necrotic cell death	9.874675325
Positive regulation of T cell receptor signaling pathway	9.874675325
Angiogenesis involved in wound healing	9.874675325
Placenta blood vessel development	9.874675325
Prostaglandin biosynthetic process	9.600378788
Immunoglobulin mediated immune response	9.600378788
Defense response to protozoan	9.425826446
Positive regulation of cytokine production	9.216363636
Leukocyte cell-cell adhesion	9.095095694
RNA catabolic process	9.095095694
I-kappaB kinase/NF-kappaB cascade	8.84127907
Cellular defense response	8.640340909
Microglial cell activation involved in immune response	8.640340909
Initiation of viral infection	8.640340909
Positive regulation of interferon-gamma biosynthetic process	8.640340909
Respiratory burst	8.640340909
Negative regulation of T cell-mediated cytotoxicity	8.640340909
N-glycan processing	8.640340909
Chylomicron remnant clearance	8.640340909
Regulation of mast cell degranulation	8.640340909
Positive regulation of interleukin-8 biosynthetic process	8.640340909
Negative regulation of nitric-oxide synthase activity	8.640340909
Maternal process involved in parturition	8.640340909
Platelet dense granule organization	8.640340909
Branching involved in embryonic placenta morphogenesis	8.640340909
Positive regulation of calcium-mediated signaling	8.064318182
Humoral immune response	8.023173701
Chemotaxis	8.018236364
Response to reactive oxygen species	7.975699301
Defense response	7.68030303
Positive regulation of T cell proliferation	7.513339921
Negative regulation of interleukin-12 production	7.406006494
Skeletal muscle tissue regeneration	7.406006494
Positive regulation of innate immune response	7.406006494
Lymph node development	7.406006494
Cellular response to lipoteichoic acid	7.406006494
Positive regulation of interleukin-1 beta secretion	7.276076555
Response to exogenous dsRNA	6.912272727
Decidualization	6.912272727
Regulation of peptidyl-tyrosine phosphorylation	6.912272727
Germinal center formation	6.912272727
Natural killer cell activation	6.912272727
Positive regulation of interleukin-17 production	6.912272727
Defense response to Gram-positive bacterium	6.646416084
Hemopoietic progenitor cell differentiation	6.583116883
JAK-STAT cascade	6.538636364
Acute inflammatory response	6.480255682
Cellular copper ion homeostasis	6.480255682
Positive regulation of B cell differentiation	6.480255682
Response to gamma radiation	6.283884298
Neutrophil chemotaxis	6.283884298
T cell activation	6.232377049
Metabolic process	6.099064171
T cell differentiation	6.099064171
Sprouting angiogenesis	6.099064171
Positive regulation of tumor necrosis factor biosynthetic process	6.099064171
Positive regulation of survival gene product expression	6.099064171
Induction of positive chemotaxis	6.099064171
Response to progesterone stimulus	6.048238636
Negative regulation of endothelial cell proliferation	5.958855799
Response to bacterium	5.760227273
Response to interferon-gamma	5.760227273
Induction of apoptosis via death domain receptors	5.760227273
Negative regulation of blood coagulation	5.760227273
Positive regulation of smooth muscle contraction	5.760227273
Inflammatory response	5.541909621
Negative regulation of growth of symbiont in host	5.529818182
Oligosaccharide metabolic process	5.457057416
Cellular component movement	5.368755516
Negative regulation of cell adhesion	5.236570248
Mesoderm development	5.236570248
Copper ion transport	5.184204545
Positive regulation of blood coagulation	5.184204545
Negative regulation of phosphorylation	5.184204545
Positive regulation of NF-kappaB import into nucleus	5.184204545
Positive regulation of blood vessel endothelial cell migration	5.184204545
Nitrogen compound metabolic process	4.937337662
Positive regulation vascular endothelial growth factor production	4.937337662
Positive regulation of interleukin-8 production	4.937337662
Response to cytokine stimulus	4.883670949
Positive regulation of interleukin-6 production	4.838590909
Response to inorganic substance	4.767084639
Response to vitamin D	4.767084639
Amino acid transport	4.712913223
B cell proliferation	4.712913223
Positive regulation of erythrocyte differentiation	4.712913223
Response to interleukin-1	4.670454545
Positive regulation of interferon-gamma production	4.564708405
Lipopolysaccharide-mediated signaling pathway	4.430944056
Positive regulation of B cell proliferation	4.412088975
Positive regulation of smooth muscle cell proliferation	4.388744589
Negative regulation of T cell proliferation	4.320170455
Response to virus	4.291934046
Ion transport	4.203409091
Protein import into nucleus, translocation	4.189256198
Negative regulation of NF-kappaB transcription factor activity	4.10050077
B cell differentiation	4.066042781
Heterophilic cell-cell adhesion	4.066042781
Response to mechanical stimulus	3.879336735
Immune response	3.769560495
Positive regulation of tumor necrosis factor production	3.75666996
Cholesterol efflux	3.736363636
B cell receptor signaling pathway	3.736363636
Induction of apoptosis	3.697923681
Phagocytosis	3.456136364
Positive regulation of nitric oxide biosynthetic process	3.388368984
Positive regulation of NF-kappaB transcription factor activity	3.352968114
Regulation of cell adhesion	3.344648094
Rho protein signal transduction	3.323208042
Positive regulation of inflammatory response	3.323208042
Anti-apoptosis	3.318806442
Response to stimulus	3.291558442
Response to peptide hormone stimulus	3.200126263
Protein complex assembly	3.19027972
Cellular calcium ion homeostasis	3.174002783
Elevation of cytosolic calcium ion concentration	3.141942149
Positive regulation of angiogenesis	3.110522727
Response to wounding	3.049532086
Cell-cell signaling	3.034012838
Induction of apoptosis by extracellular signals	3.005335968
Defense response to virus	3.005335968
Response to lipopolysaccharide	2.948362775
Positive regulation of peptidyl-tyrosine phosphorylation	2.94139265
Protein homooligomerization	2.928929122
Cellular amino acid metabolic process	2.840660025
Positive regulation of I-kappaB kinase/NF-kappaB cascade	2.757555609
Cell adhesion	2.737533753
Fatty acid biosynthetic process	2.728528708
Interspecies interaction between organisms	2.696985024
Osteoblast differentiation	2.69309327
Transmembrane receptor protein tyrosine kinase signaling pathway	2.546626794
Signal transduction	2.529953417
Activation of MAPK activity	2.449230494
Innate immune response	2.367216687
Skeletal system development	2.319554606
Response to oxidative stress	2.273773923
Response to hypoxia	2.15335599
Cell proliferation	2.122933884
Cell death	2.057224026
Multicellular organismal development	1.901434245
Negative regulation of cell proliferation	1.840545992
Lipid metabolic process	1.78765674
Positive regulation of transcription from RNA polymerase II promoter	0.643002114
Transmembrane transport	0.599113562
Small GTPase-mediated signal transduction	0.433824648
Regulation of transcription, DNA-dependent	0.407975625
Intracellular protein transport	0.399553337
Axon guidance	0.360014205
Translation	0.261432403
DNA repair	0.23247554
Antigen processing and presentation of peptide antigen via MHC class I	0.174552342
Positive regulation of transcription, DNA-dependent	0.17004361
Protein folding	0.164970709
Protein ubiquitination	0.154291802
mRNA processing	0.152926388
Xenobiotic metabolic process	0.12042287
Synaptic transmission	0.116172651
Negative regulation of transcription, DNA-dependent	0.112945633
Mitotic cell cycle	0.104100493
Fibroblast growth factor receptor signaling pathway	0.101950925
Transcription, DNA-dependent	0.016632033
Transcription, DNA-dependent	−0.039880623
Translation	−0.062686789
Blood coagulation	−0.076875635
Intracellular protein transport	−0.079838087
DNA repair	−0.092905756
Regulation of small GTPase-mediated signal transduction	−0.160604524
Viral reproduction	−0.162175997
Innate immune response	−0.227046396
Mitotic cell cycle	−0.249614261
Positive regulation of transcription, DNA-dependent	−0.254833747
Cytokine-mediated signaling pathway	−0.264766564
Negative regulation of transcription, DNA-dependent	−0.270823316
Immune response	−0.355455602
Positive regulation of transcription from RNA polymerase II promoter	−0.385450859
Negative regulation of transcription from RNA polymerase II promoter	−0.389854531
Small GTPase-mediated signal transduction	−0.404535246
Protein transport	−0.408638731
Proteolysis	−0.541854957
Regulation of transcription, DNA-dependent	−0.628876401
Transport	−1.651693671
Signal transduction	−1.661658778
Positive regulation of cell proliferation	−1.826982685
Response to ethanol	−2.273925035
Regulation of cell shape	−2.419618529
Cell differentiation	−2.426430518
Kidney development	−2.458065857
Cell-cell signaling	−2.530440751
Homophilic cell adhesion	−2.617008461
Muscle organ development	−2.736337463
Neuron differentiation	−2.825179589
Chromatin modification	−2.900517711
Lipid catabolic process	−2.95971195
Regulation of cell growth	−2.986376022
Sodium ion transport	−2.99537113
Calcium ion transport	−3.107697548
Inner ear morphogenesis	−3.139088432
Fatty acid metabolic process	−3.1873821
Response to stimulus	−3.288568834
Palate development	−3.333928404
Neuron projection morphogenesis	−3.341610266
Potassium ion transport	−3.452997275
Transmembrane receptor protein tyrosine kinase signaling pathway	−3.489344615
Cellular response to insulin stimulus	−3.489344615
Memory	−3.511522653
Response to hormone stimulus	−3.511522653
Nervous system development	−3.540414928
Cell adhesion	−3.575097603
Embryonic digit morphogenesis	−3.710683639
Muscle contraction	−3.732970027
Positive regulation of glucose import	−3.9462826
Learning or memory	−4.062349736
Synapse assembly	−4.14359673
Odontogenesis	−4.14359673
Multicellular organismal development	−4.157006428
Positive regulation of canonical Wnt receptor signaling pathway	−4.213827183
Behavior	−4.2498428
Regulation of heart contraction	−4.361680769
Response to morphine	−4.361680769
Neurogenesis	−4.479564033
Negative regulation of insulin secretion	−4.603996367
Negative regulation of epithelial cell proliferation	−4.73553912
Branching morphogenesis of a tube	−4.73553912
Embryonic skeletal system development	−4.93285325
Positive regulation of mesenchymal cell proliferation	−4.972316076
Tissue regeneration	−5.022541491
Negative regulation of Wnt receptor signaling pathway	−5.179495913
Central nervous system development	−5.217862549
Neurotransmitter transport	−5.404691387
Retinal ganglion cell axon guidance	−5.404691387
Positive regulation of blood pressure	−5.404691387
Calcium-dependent cell-cell adhesion	−5.452100961
Activation of phospholipase C activity by G-protein coupled receptor protein signaling Pathway coupled to IP3 second messenger	−5.599455041
Middle ear morphogenesis	−5.715305835
Eye development	−6.542521153
Positive regulation of epithelial cell proliferation	−6.605733918
Positive regulation of cell differentiation	−6.683220533
Mammary gland development	−6.90599455
Regulation of smooth muscle contraction	−6.90599455
Positive regulation of tyrosine phosphorylation of Stat5 protein	−6.90599455
Hemopoietic stem cell proliferation	−6.90599455
Neural tube development	−7.206255183
Calcium ion transport into cytosol	−7.312229524
Mesonephros development	−7.769243869
Peptide cross-linking via chondroitin 4-sulfate glycosaminoglycan	−7.769243869
Prostate gland growth	−7.769243869
Ion transport	−8.119209809
Regulation of synaptic transmission	−8.28719346
Positive regulation of insulin-like growth factor receptor signaling pathway	−8.28719346
Positive regulation of cyclin-dependent protein kinase activity	−8.28719346
Vagina development	−8.28719346
Cardiac muscle tissue development	−8.879135851
Transmembrane receptor protein tyrosine phosphatase signaling pathway	−9.207992734
Choline metabolic process	−9.207992734
Negative regulation of epinephrine secretion	−9.207992734
Protein insertion into membrane	−9.207992734
Type II pneumocyte differentiation	−9.207992734
Activation of protein kinase B activity	−9.562146301
Metabolic process	−9.749639365
Regulation of respiratory gaseous exchange by neurological system process	−10.35899183
Creatine metabolic process	−10.35899183
Retinal metabolic process	−10.35899183
Peptide biosynthetic process	−10.35899183
Growth hormone receptor signaling pathway	−10.35899183
Hormone-mediated signaling pathway	−11.30071836
Rhythmic process	−11.50999092
Cardiac left ventricle morphogenesis	−11.8388478
Positive regulation of cyclin-dependent protein kinase activity involved in G1/S	−11.8388478
Positive regulation of lymphocyte proliferation	−11.8388478
Ovulation cycle	−13.8119891
Negative regulation of norepinephrine secretion	−13.8119891
Taurine metabolic process	−13.8119891
Negative regulation of actin filament polymerization	−13.8119891
Positive regulation of potassium ion transport	−13.8119891
Urinary bladder development	−13.8119891
Morphogenesis of an epithelial fold	−13.8119891
Ciliary neurotrophic factor-mediated signaling pathway	−13.8119891
Aromatic compound catabolic process	−16.57438692
Negative regulation of phagocytosis	−16.57438692
Tertiary branching involved in mammary gland duct morphogenesis	−16.57438692
Cellular response to heparin	−16.57438692
Regulation of vasodilation	−17.7582717
Nucleoside triphosphate catabolic process	−20.71798365
Saliva secretion	−20.71798365
Female genitalia morphogenesis	−20.71798365
Smooth muscle contraction involved in micturition	−20.71798365
Regulation of prostatic bud formation	−20.71798365
Pericardium morphogenesis	−27.6239782
Lateral sprouting involved in mammary gland duct morphogenesis	−27.6239782
Regulation of protein metabolic process	−41.4359673
Negative regulation of the force of heart contraction involved in baroreceptor response to Increased systemic arterial blood pressure	−41.4359673
Renin secretion into blood stream	−41.4359673
Regulation of thyroid hormone mediated signaling pathway	−41.4359673
Negative regulation of leukocyte chemotaxis	−41.4359673
Pyruvate transport	−41.4359673
Nucleoside diphosphate catabolic process	−41.4359673
Glycolate metabolic process	−41.4359673
Negative regulation of lamellipodium assembly	−41.4359673
Muscle hypertrophy	−41.4359673
Myotube cell development	−41.4359673
Mevalonate transport	−41.4359673
Male somatic sex determination	−41.4359673
Spinal cord patterning	−41.4359673
Orbitofrontal cortex development	−41.4359673
Cell-cell adhesion involved in neuronal-glial interactions involved in cerebral cortex radial glia guided migration	−41.4359673
Corticospinal neuron axon guidance through spinal cord	−41.4359673
Neural plate mediolateral regionalization	−41.4359673
Cellular potassium ion homeostasis	−41.4359673
Regulation of mismatch repair	−41.4359673
Positive regulation of phospholipase A2 activity	−41.4359673
Retinol transport	−41.4359673
Response to luteinizing hormone stimulus	−41.4359673
Positive regulation of locomotion	−41.4359673
Sequestering of neurotransmitter	−41.4359673
Carnitine catabolic process	−41.4359673
Homocysteine catabolic process	−41.4359673
Regulation of adenylate cyclase activity	−41.4359673
Phosphatidic acid metabolic process	−41.4359673
Regulation of saliva secretion	−41.4359673
Paraxial mesoderm structural organization	−41.4359673
Intermediate mesoderm development	−41.4359673
Urothelial cell proliferation	−41.4359673
Induction of negative chemotaxis	−41.4359673
Regulation of lipid catabolic process	−41.4359673
Negative regulation of small GTPase-mediated signal transduction	−41.4359673
Micturition	−41.4359673
Activation of prostate induction by androgen receptor signaling pathway	−41.4359673
Neural plate pattern specification	−41.4359673
Dermatome development	−41.4359673
Negative regulation of activation-induced cell death of T cells	−41.4359673
Cellular response to chemical stimulus	−41.4359673
Negative regulation of smooth muscle cell chemotaxis	−41.4359673
Negative regulation of mononuclear cell migration	−41.4359673
Pattern specification involved in metanephros development	−41.4359673
Negative regulation of neutrophil chemotaxis	−41.4359673
Metanephric cap mesenchymal cell proliferation involved in metanephros development	−41.4359673
Positive regulation of non-canonical Wnt receptor signaling pathway	−41.4359673
Positive regulation of Wnt receptor signaling pathway involved in dorsal/ventral axis specification	−41.4359673

**Table V:** Upregulated significant pathways.

Pathway name	-LgP
Cytokine-cytokine receptor interaction	23.42004685
Leishmaniasis	21.90107349
Cell adhesion molecules (CAMs)	20.65682392
Chagas disease	15.37890961
T cell receptor signaling pathway	13.94901928
Hematopoietic cell lineage	13.74068504
Natural killer cell mediated cytotoxicity	13.17649459
Chemokine signaling pathway	13.14867922
Primary immunodeficiency	13.11509338
Phagosome	12.87858647
Antigen processing and presentation	12.1066584
Intestinal immune network for IgA production	12.08192561
Malaria	11.93017145
Toll-like receptor signaling pathway	11.56038144
Allograft rejection	11.39679734
Graft-versus-host disease	11.05163889
B cell receptor signaling pathway	10.95069165
Type I diabetes mellitus	10.72782064
Viral myocarditis	10.43654793
NOD-like receptor signaling pathway	9.858981253
Asthma	8.781008205
Jak-STAT signaling pathway	8.780882537
Leukocyte transendothelial migration	8.69910665
Amoebiasis	8.385599375
Systemic lupus erythematosus	8.176130358
Autoimmune thyroid disease	8.170383587
Fc gamma R-mediated phagocytosis	7.492623136
Fc epsilon RI signaling pathway	6.80371883
Complement and coagulation cascades	6.801648207
Apoptosis	5.705278343
Lysosome	5.334893435
Pathways in cancer	4.192720575
Regulation of actin cytoskeleton	4.003028031
p53 signaling pathway	3.535108478
Cytosolic DNA-sensing pathway	3.092429362
Neuroactive ligand-receptor interaction	3.078524076
Calcium signaling pathway	2.822547274
Epithelial cell signaling in *Helicobacter pylori* infection	2.818702804
MAPK signaling pathway	2.752907643
RIG-I-like receptor signaling pathway	2.693488553
Neurotrophin signaling pathway	2.625676671
Pantothenate and CoA biosynthesis	2.610488424
Acute myeloid leukemia	2.603797719
Arachidonic acid metabolism	2.514704889
Focal adhesion	2.36365754
TGF-beta signaling pathway	2.223171679
Prion diseases	2.198269973
Olfactory transduction	2.139616901
mTOR signaling pathway	2.121221511
Amyotrophic lateral sclerosis (ALS)	2.079595295
Endocytosis	1.906955325
VEGF signaling pathway	1.890777366
Aldosterone-regulated sodium reabsorption	1.851879925
Shigellosis	1.780192486
Glycosaminoglycan biosynthesis - keratan sulfate	1.771193081
Small cell lung cancer	1.633206157
Other glycan degradation	1.615330588
Salivary secretion	1.530885213
Renal cell carcinoma	1.501166586
Pancreatic cancer	1.501166586
Melanoma	1.473244623
Ether lipid metabolism	1.40495844

-LgP, negative logarithm of the P-value.

**Table VI:** Pathways associated with significantly downregulated genes.

Pathway name	-LgP
Focal adhesion	8.1140073
Neuroactive ligand-receptor interaction	5.712988
Arrhythmogenic right ventricular cardiomyopathy (ARVC)	5.5013372
Calcium signaling pathway	5.445408
Wnt signaling pathway	5.1186354
Vascular smooth muscle contraction	5.0207839
Long-term depression	4.8678343
Aldosterone-regulated sodium reabsorption	3.9213362
Axon guidance	3.8465848
Dilated cardiomyopathy	3.8147938
Hypertrophic cardiomyopathy (HCM)	3.4512102
ErbB signaling pathway	3.2950005
Salivary secretion	3.2203532
Adherens junction	3.1261703
Pathways in cancer	3.0889176
Pancreatic secretion	2.8145968
Glycine, serine and threonine metabolism	2.7224771
Glioma	2.7174562
ECM-receptor interaction	2.6818514
Progesterone-mediated oocyte maturation	2.6485594
Glycerolipid metabolism	2.6032586
Long-term potentiation	2.5263793
Regulation of actin cytoskeleton	2.3885129
Tyrosine metabolism	2.3264949
Phosphatidylinositol signaling system	2.2235491
GnRH signaling pathway	2.204073
Melanogenesis	2.204073
Nicotinate and nicotinamide metabolism	2.1809844
ABC transporters	2.093767
Olfactory transduction	2.080365
Histidine metabolism	2.0047222
Cell adhesion molecules (CAMs)	1.9933196
Oocyte meiosis	1.9319734
MAPK signaling pathway	1.9265796
Gap junction	1.9104997
Insulin signaling pathway	1.8902518
PPAR signaling pathway	1.8327772
Melanoma	1.8327772
Fc gamma R-mediated phagocytosis	1.7609805
Phenylalanine metabolism	1.7586389
Gastric acid secretion	1.7471867
Arginine and proline metabolism	1.6184534
Inositol phosphate metabolism	1.5877798
Circadian rhythm - mammal	1.5137608
Tryptophan metabolism	1.5097806
Amoebiasis	1.4980899
Chemokine signaling pathway	1.4705506
Purine metabolism	1.4329084
Prostate cancer	1.3808613
Fatty acid metabolism	1.3705322
Valine, leucine and isoleucine degradation	1.3705322

-LgP, negative logarithm of the P-value.

**Table VII:** Characteristics of genes.

Gene symbol	Description	Betweenness centrality	Degree	Indegree	Outdegree	Style
JAK3	Janus kinase 3	0.038452356	31	26	5	Up
GNA15	Guanine nucleotide binding protein (G protein), alpha 15 (Gq class)	0.037599143	11	8	3	Up
MAPK13	Mitogen-activated protein kinase 13	0.033950795	9	6	3	Up
ARRB2	Arrestin, beta 2	0.033861892	8	7	8	Up
F2R	Coagulation factor II (thrombin) receptor	0.032585932	3	2	2	Up
VCAM1	Vascular cell adhesion molecule 1	0.031990127	7	2	5	Up
F11R	F11 receptor	0.03029902	6	6	3	Up
MLLT4	Myeloid/lymphoid or mixed-lineage leukemia (trithorax homolog, Drosophila); translocated to, 4	0.028330801	4	4	4	Down
STAT1	Signal transducer and activator of transcription 1, 91 kDa	0.027727017	7	2	5	Up
ACTN2	Actinin, alpha 2	0.027721674	5	5	5	Down
SSX2IP	Synovial sarcoma, X breakpoint 2 interacting protein	0.026250152	2	2	2	Down
PRKCB	Protein kinase C, beta	0.02479466	12	5	8	Down
PRKCA	Protein kinase C, alpha	0.024281711	11	5	10	Up
PRKX	Protein kinase, X-linked	0.018822441	12	4	9	Up
ITGA4	Integrin, alpha 4 (antigen CD49D, alpha 4 subunit of VLA-4 receptor)	0.016703185	15	14	3	Up
SOCS3	Suppressor of cytokine signaling 3	0.01636563	21	2	19	Up
ITGA9	Integrin, alpha 9	0.016034992	14	13	3	Down
CXCL1	Chemokine (C-X-C motif) ligand 1 (melanoma growth stimulating activity, alpha)	0.015128782	7	1	6	Up
IL8	Interleukin 8	0.015128782	7	1	6	Up
NCF4	Neutrophil cytosolic factor 4, 40 kDa	0.014984515	2	1	1	Up
CYBB	Cytochrome b-245, beta polypeptide	0.014984515	2	1	1	Up
CXCL10	Chemokine (C-X-C motif) ligand 10	0.014593392	7	1	6	Up
PLCB4	Phospholipase C, beta 4	0.01422471	7	6	2	Down
PLCB1	Phospholipase C, beta 1 (phosphoinositide-specific)	0.01422471	7	6	2	Down
PLCB2	Phospholipase C, beta 2	0.01422471	7	6	2	Up
LYN	v-yes-1 Yamaguchi sarcoma viral related oncogene homolog	0.011954016	20	3	17	Up
GRIA2	Glutamate receptor, ionotropic, AMPA 2	0.010419088	3	1	2	Down
STAT4	Signal transducer and activator of transcription 4	0.008717991	4	1	3	Up
CCR7	Chemokine (C-C motif) receptor 7	0.008388993	12	11	2	Up
CCR4	Chemokine (C-C motif) receptor 4	0.008388993	12	11	2	Up
CCR2	Chemokine (C-C motif) receptor 2	0.008388993	12	11	2	Up
CX3CR1	Chemokine (C-X3-C motif) receptor 1	0.008388993	12	11	2	Up
CCR1	Chemokine (C-C motif) receptor 1	0.008388993	12	11	2	Up
CXCR4	Chemokine (C-X-C motif) receptor 4	0.008388993	12	11	2	Up
CSF2RA	Colony stimulating factor 2 receptor, alpha, low-affinity (granulocyte-macrophage)	0.005161547	4	3	2	Up
ITGB2	Integrin, beta 2 (complement component 3 receptor 3 and 4 subunit)	0.004818256	7	4	5	Up
SPI1	Spleen focus forming virus (SFFV) proviral integration oncogene spi1	0.003699643	3	3	2	Up
PGF	Placental growth factor	0.003543193	5	2	3	Up
GRIN2A	Glutamate receptor, ionotropic, N-methyl D-aspartate 2A	0.003531713	5	5	2	Down
VAV1	Vav 1 guanine nucleotide exchange factor	0.002788411	11	11	5	Up
RYR2	Ryanodine receptor 2 (cardiac)	0.002768397	7	7	6	Down
NFATC2	Nuclear factor of activated T-cells, cytoplasmic, calcineurin-dependent 2	0.002250563	3	1	2	Up
SYK	Spleen tyrosine kinase	0.002031231	17	8	12	Up
PIK3CG	Phosphoinositide-3-kinase, catalytic, gamma polypeptide	0.001985436	23	21	5	Up
PIK3R5	Phosphoinositide-3-kinase, regulatory subunit 5	0.001985436	23	21	5	Up
PIK3R1	Phosphoinositide-3-kinase, regulatory subunit 1 (alpha)	0.001985436	23	21	5	Down
ITGAL	Integrin, alpha L [antigen CD11A (p180), lymphocyte function-associated antigen 1; alpha polypeptide]	0.001811094	6	3	4	Up
FZD3	Frizzled homolog 3 (Drosophila)	0.001625406	8	4	5	Down
FZD4	Frizzled homolog 4 (Drosophila)	0.001625406	8	4	5	Down
PTPN6	Protein tyrosine phosphatase, non-receptor type 6	0.001615789	26	2	26	Up
PDE3B	Phosphodiesterase 3B, cGMP-inhibited	0.001519611	2	2	2	Down
THY1	Thy-1 cell surface antigen	0.001513199	2	2	2	Up
PIP5K1B	Phosphatidylinositol-4-phosphate 5-kinase, type I, beta	0.001487551	3	1	3	Down
LCK	Lymphocyte-specific protein tyrosine kinase	0.001429845	4	3	3	Up
CSF1R	Colony stimulating factor 1 receptor	0.001429845	3	3	2	Up
RYR3	Ryanodine receptor 3	0.001408472	6	6	6	Down
TYROBP	TYRO protein tyrosine kinase binding protein	0.001384962	2	1	1	Up
IFNAR2	Interferon (alpha, beta and omega) receptor 2	0.001350765	4	2	2	Up
LCP2	Lymphocyte cytosolic protein 2 (SH2 domain containing leukocyte protein of 76 kDa)	0.001191975	6	5	4	Up
CD4	CD4 molecule	0.001077192	9	9	1	Up
PVRL2	Poliovirus receptor-related 2 (herpesvirus entry mediator B)	0.000865601	3	1	3	Up
FCGR3A	Fc fragment of IgG, low affinity IIIa, receptor (CD16a)	0.00085349	5	5	3	Up
CCL5	Chemokine (C-C motif) ligand 5	0.000801482	7	1	6	Up
SORBS1	Sorbin and SH3 domain containing 1	0.000795071	2	2	1	Down
TNF	Tumor necrosis factor	0.000756599	2	1	1	Up
EGFR	Epidermal growth factor receptor	0.000745058	15	10	5	Down
ITK	IL2-inducible T-cell kinase	0.00071535	8	7	2	Up
ITGA7	Integrin, alpha 7	0.000649735	13	13	2	Down
PDGFRB	Platelet-derived growth factor receptor, beta polypeptide	0.00059812	12	7	5	Up
KDR	Kinase insert domain receptor (a type III receptor tyrosine kinase)	0.000530261	10	5	5	Down
BTK	Bruton agammaglobulinemia tyrosine kinase	0.000391704	6	6	4	Up
CACNB2	Calcium channel, voltage-dependent, beta 2 subunit	0.000371888	2	2	1	Down
CACNA2D3	Calcium channel, voltage-dependent, alpha 2/delta subunit 3	0.000371888	2	2	1	down
IFNGR2	Interferon gamma receptor 2 (interferon gamma transducer 1)	0.000368682	4	2	2	Up
IFNGR1	Interferon gamma receptor 1	0.000368682	4	2	2	Up
FLNC	Filamin C, gamma	0.00036227	3	3	3	Down
CACNA1H	Calcium channel, voltage-dependent, T type, alpha 1H subunit	0.000281511	3	3	3	Up
PARD3	Par-3 partitioning defective 3 homolog (C. elegans)	0.000278053	3	2	1	Down
RASGRP1	RAS guanyl releasing protein 1 (calcium and DAG-regulated)	0.000218003	3	3	3	Up
CD8A	CD8a molecule	0.000134649	2	2	1	Up
IGF1	Insulin-like growth factor 1 (somatomedin C)	0.000121825	10	1	9	Down
HIF1A	Hypoxia inducible factor 1, alpha subunit (basic helix-loop-helix transcription factor)	0.000115413	3	1	3	Up
FGFR2	Fibroblast growth factor receptor 2	0.000105796	9	6	3	Down
CSF2RB	Colony stimulating factor 2 receptor, beta, low-affinity (granulocyte-macrophage)	0.000100025	6	2	4	Up
GNAO1	Guanine nucleotide binding protein (G protein), alpha activating activity polypeptide O	7.69423E-05	7	2	5	Down
CD28	CD28 molecule	7.69423E-05	5	1	4	Up
ERBB4	v-erb - a erythroblastic leukemia viral oncogene homolog 4 (avian)	6.35843E-05	7	2	5	Down
BLNK	B-cell linker	5.79918E-05	3	3	2	Up
HCK	Hemopoietic cell kinase	4.51375E-05	10	1	9	Up
PLCE1	Phospholipase C, epsilon 1	2.88534E-05	2	2	2	Down
HLA-DMB	Major histocompatibility complex, class II, DM beta	1.92356E-05	9	7	9	Up
HLA-DMA	Major histocompatibility complex, class II, DM alpha	1.92356E-05	9	7	9	Up
PIK3AP1	Phosphoinositide-3-kinase adaptor protein 1	1.28237E-05	4	4	3	Up
LRP6	Low density lipoprotein receptor-related protein 6	1.28237E-05	2	2	2	Down
CD247	CD247 molecule	9.61779E-06	3	2	2	Up
ITGAM	Integrin, alpha M (complement component 3 receptor 3 subunit)	8.54915E-06	3	2	2	Up
INHBA	Inhibin, beta A	6.41186E-06	2	1	1	Up
TRPC1	Transient receptor potential cation channel, subfamily C, member 1	5.80121E-06	2	2	2	Down
BST1	Bone marrow stromal cell antigen 1	5.80121E-06	2	2	2	Up
P2RX4	Purinergic receptor P2X, ligand-gated ion channel, 4	5.80121E-06	2	2	2	Up
P2RX1	Purinergic receptor P2X, ligand-gated ion channel, 1	5.80121E-06	2	2	2	Up
FCGR2A	Fc fragment of IgG, low affinity IIa, receptor (CD32)	3.91836E-06	3	3	1	Up
PRKAR2B	Protein kinase, cAMP-dependent, regulatory, type II, beta	3.20593E-06	2	1	2	Down
SHC3	SHC (Src homology 2 domain containing) transforming protein 3	0	9	9	0	Down
SHC2	SHC (Src homology 2 domain containing) transforming protein 2	0	9	9	0	Down
HLA-DQA2	Major histocompatibility complex, class II, DQ alpha 2	0	8	7	8	Up
HLA-DPA1	Major histocompatibility complex, class II, DP alpha 1	0	8	7	8	Up
HLA-DRA	Major histocompatibility complex, class II, DR alpha	0	8	7	8	Up
HLA-DPB1	Major histocompatibility complex, class II, DP beta 1	0	8	7	8	Up
HLA-DOA	Major histocompatibility complex, class II, DO alpha	0	8	7	8	Up
HLA-DOB	Major histocompatibility complex, class II, DO beta	0	8	7	8	Up
RXRG	Retinoid X receptor, gamma	0	7	0	7	Down
FGR	Gardner-Rasheed feline sarcoma viral (v-fgr) oncogene homolog	0	7	0	7	Up
GNG7	Guanine nucleotide binding protein (G protein), gamma 7	0	6	0	6	Down
CXCL2	Chemokine (C-X-C motif) ligand 2	0	6	0	6	Up
CCL4L1	Chemokine (C-C motif) ligand 4-like 1	0	6	0	6	Up
CCL3	Chemokine (C-C motif) ligand 3	0	6	0	6	Up
CCL2	Chemokine (C-C motif) ligand 2	0	6	0	6	Up
CXCL14	Chemokine (C-X-C motif) ligand 14	0	6	0	6	Down
CXCL16	Chemokine (C-X-C motif) ligand 16	0	6	0	6	Up
WWP1	WW domain containing E3 ubiquitin protein ligase 1	0	5	0	5	Down
CD74	CD74 molecule, major histocompatibility complex, class II invariant chain	0	5	5	0	Up
PGR	Progesterone receptor	0	4	0	4	Down
PLA2G2A	Phospholipase A2, group IIA (platelets, synovial fluid)	0	4	4	0	Down
FCER1G	Fc fragment of IgE, high affinity I, receptor for; gamma polypeptide	0	4	3	3	Up
CCND2	Cyclin D2	0	4	4	0	Down
PLA2G5	Phospholipase A2, group V	0	4	4	0	Up
RASSF5	Ras association (RalGDS/AF-6) domain family member 5	0	4	0	4	Up
PDGFA	Platelet-derived growth factor alpha polypeptide	0	4	0	4	Up
WNT5A	Wingless-type MMTV integration site family, member 5A	0	4	2	2	Down
LIFR	Leukemia inhibitory factor receptor alpha	0	3	2	1	Down
CD226	CD226 molecule	0	3	3	0	Up
LAMB3	Laminin, beta 3	0	3	0	3	Up
SPP1	Secreted phosphoprotein 1	0	3	0	3	Up
HGF	Hepatocyte growth factor (hepapoietin A; scatter factor)	0	3	0	3	Up
LAMA2	Laminin, alpha 2	0	3	0	3	Down
SFRP1	Secreted frizzled-related protein 1	0	3	0	3	Down
SFRP2	Secreted frizzled-related protein 2	0	3	0	3	Down
FGF9	Fibroblast growth factor 9 (glia-activating factor)	0	3	0	3	Down
IL13RA1	Interleukin 13 receptor, alpha 1	0	3	2	1	Up
GHR	Growth hormone receptor	0	3	2	1	Down
ICAM1	Intercellular adhesion molecule 1	0	3	3	0	Up
IL6R	Interleukin 6 receptor	0	3	2	1	Up
IL7R	Interleukin 7 receptor	0	3	2	1	Up
AMPH	Amphiphysin	0	3	3	0	Down
COL6A6	Collagen, type VI, alpha 6	0	3	0	3	Down
IL10RB	Interleukin 10 receptor, beta	0	3	2	1	Up
THBS1	Thrombospondin 1	0	3	0	3	Up
THBS2	Thrombospondin 2	0	3	0	3	Up
THBS4	Thrombospondin 4	0	3	0	3	Down
FGF10	Fibroblast growth factor 10	0	3	0	3	Down
TGFB1	Transforming growth factor, beta 1	0	3	3	0	Up
FGF20	Fibroblast growth factor 20	0	3	0	3	Up
IL11RA	Interleukin 11 receptor, alpha	0	3	2	1	Down
HCST	Hematopoietic cell signal transducer	0	3	0	3	Up
RELN	Reelin	0	3	0	3	Down
COL1A1	Collagen, type I, alpha 1	0	3	0	3	Up
CNTFR	Ciliary neurotrophic factor receptor	0	3	2	1	Down
DOCK2	Dedicator of cytokinesis 2	0	3	3	0	Up
IL12RB1	Interleukin 12 receptor, beta 1	0	3	2	1	Up
ICOS	Inducible T-cell co-stimulator	0	3	0	3	Up
MYLK	Myosin light chain kinase	0	3	3	0	Down
IL21R	Interleukin 21 receptor	0	3	2	1	Up
PAK7	p21 protein (Cdc42/Rac)-activated kinase 7	0	3	0	3	Down
PAK3	p21 protein (Cdc42/Rac)-activated kinase 3	0	3	0	3	Down
IL2RG	Interleukin 2 receptor, gamma	0	3	2	1	Up
PTPRC	Protein tyrosine phosphatase, receptor type, C	0	3	0	3	Up
IL2RB	Interleukin 2 receptor, beta	0	3	2	1	Up
TNXB	Tenascin XB	0	3	0	3	Down
IL2RA	Interleukin 2 receptor, alpha	0	3	2	1	Up
COL4A6	Collagen, type IV, alpha 6	0	3	0	3	Down
BTC	Betacellulin	0	2	0	2	Down
FAS	Fas (TNF receptor superfamily, member 6)	0	2	2	0	Up
PTPRF	Protein tyrosine phosphatase, receptor type, F	0	2	0	2	Down
PIM1	Pim-1 oncogene	0	2	2	0	Up
GNAZ	Guanine nucleotide binding protein (G protein), alpha z polypeptide	0	2	0	2	Down
KCNMA1	Potassium large conductance calcium-activated channel, subfamily M, alpha member 1	0	2	2	0	Down
GZMA	Granzyme A (granzyme 1, cytotoxic T-lymphocyte-associated serine esterase 3)	0	2	0	2	Up
ARHGEF6	Rac/Cdc42 guanine nucleotide exchange factor (GEF) 6	0	2	2	0	Up
ICAM3	Intercellular adhesion molecule 3	0	2	0	2	Up
MMP14	Matrix metallopeptidase 14 (membrane-inserted)	0	2	2	0	Up
RUNX1	Runt-related transcription factor 1	0	2	1	2	Up
LIMK1	LIM domain kinase 1	0	2	2	0	Up
LIPE	Lipase, hormone-sensitive	0	2	2	0	Down
PTGS2	Prostaglandin-endoperoxide synthase 2 (prostaglandin G/H synthase and cyclooxygenase)	0	1	1	0	Up
ADCY5	Adenylate cyclase 5	0	1	0	1	Down
FST	Follistatin	0	1	0	1	Up
LHCGR	Luteinizing hormone/choriogonadotropin receptor	0	1	0	1	Down
PRKG1	Protein kinase, cGMP-dependent, type I	0	1	0	1	Down
AGTR1	Angiotensin II receptor, type 1	0	1	0	1	Down
PPP1R1A	Protein phosphatase 1, regulatory (inhibitor) subunit 1A	0	1	1	0	Down
MAP2K6	Mitogen-activated protein kinase kinase 6	0	1	0	1	Down
LILRB3	Leukocyte immunoglobulin-like receptor, subfamily B (with TM and ITIM domains), member 3	0	1	1	1	Up
GRAP2	GRB2-related adaptor protein 2	0	1	1	1	Up
DUSP10	Dual specificity phosphatase 10	0	1	0	1	Up
CD72	CD72 molecule	0	1	1	1	Up
CTSL1	Cathepsin L1	0	1	0	1	Up
AVPR1A	Arginine vasopressin receptor 1A	0	1	0	1	Down
AREG	Amphiregulin	0	1	0	1	Up
WAS	Wiskott-Aldrich syndrome (eczema-thrombocytopenia)	0	1	1	0	Up
CARD11	Caspase recruitment domain family, member 11	0	1	1	0	Up
DAPP1	Dual adaptor of phosphotyrosine and 3-phosphoinositides	0	1	1	0	Up
PLIN1	Perilipin 1	0	1	1	0	Down
GRIA3	Glutamate receptor, ionotrophic, AMPA 3	0	1	0	1	Down
RPS6KA6	Ribosomal protein S6 kinase, 90kDa, polypeptide 6	0	1	1	0	Down
DUSP2	Dual specificity phosphatase 2	0	1	0	1	Up
RPS6KA1	Ribosomal protein S6 kinase, 90kDa, polypeptide 1	0	1	1	0	Up
ARNT2	Aryl-hydrocarbon receptor nuclear translocator 2	0	1	1	1	Down
CDKN2B	Cyclin-dependent kinase inhibitor 2B (p15, inhibits CDK4)	0	1	0	1	Up
CDKN2C	Cyclin-dependent kinase inhibitor 2C (p18, inhibits CDK4)	0	1	0	1	Up
MS4A2	Membrane-spanning 4-domains, subfamily A, member 2 (Fc fragment of IgE, high affinity I, receptor for; beta polypeptide)	0	1	1	0	Up
HLA-E	Major histocompatibility complex, class I, E	0	1	0	1	Up
CYSLTR2	Cysteinyl leukotriene receptor 2	0	1	0	1	Up
ANGPTL4	Angiopoietin-like 4	0	1	1	0	Up
FABP4	Fatty acid binding protein 4, adipocyte	0	1	1	0	Down
CD79A	CD79a molecule, immunoglobulin-associated alpha	0	1	1	0	Up
ADRA1D	Adrenergic, alpha-1D-, receptor	0	1	0	1	Down
TACR1	Tachykinin receptor 1	0	1	0	1	Down
GSN	Gelsolin	0	1	1	0	Down
EGR1	Early growth response 1	0	1	0	1	Up
CTSS	Cathepsin S	0	1	0	1	Up
ACADL	Acyl-CoA dehydrogenase, long chain	0	1	1	0	Down
CD86	CD86 molecule	0	1	0	1	Up
CD80	CD80 molecule	0	1	1	0	Up
CTSB	Cathepsin B	0	1	0	1	Up
OXTR	Oxytocin receptor	0	1	0	1	Up
DCN	Decorin	0	1	0	1	Down
TNFRSF1B	Tumor necrosis factor receptor superfamily, member 1B	0	1	1	0	Up
OLR1	Oxidized low density lipoprotein (lectin-like) receptor 1	0	1	1	0	Up
OR51E2	Olfactory receptor, family 51, subfamily E, member 2	0	1	1	0	Up
ADIPOQ	Adiponectin, C1Q and collagen domain containing	0	1	1	0	Down
IGFBP3	Insulin-like growth factor binding protein 3	0	1	0	1	Up
